# Predicting Continued Participation in Government-Initiated Public Sports Clubs: The Role of Prior Knowledge and Awareness through Health Communications

**DOI:** 10.3390/ijerph18137168

**Published:** 2021-07-04

**Authors:** Doyeon Won, Hyung-Hoon Kim, Jung-Sup Bae

**Affiliations:** 1 Department of Kinesiology, Texas A&M University-Corpus Christi, Corpus Christi, TX 78412, USA; doyeon.won@tamucc.edu; 2 Department of Taekwondo and Security, Honam University, Gwangju 62399, Korea; 3 Division of Academic Affairs, Glocal Campus, Konkuk University, Chungju 27478, Korea

**Keywords:** physical activity, public sport clubs, model of goal-directed behavior, health communication, health promotion

## Abstract

Physical activity is the most effective preventive medicine in enhancing our physical health and subjective wellbeing. Since 2013, the South Korean government has introduced and developed the public sports club system as a way to promote exercise and the health of the general public. The current study investigated factors underlying the general public’s desires and intentions to join or participate in a public sports club (PSC) using the model of goal-directed behavior (MGB). Data were collected from 254 college students who had prior experience of participating in at least one PSC and were primarily analyzed using structural equation modeling (SEM). The results suggest that, among the five MGB determinants, the positive anticipated emotions and perceived behavioral control were significantly associated with participants’ desires, and, in turn, their desires were significantly related to their intention to participate in PSCs. Meanwhile, the respondents’ prior experience was marginally but significantly associated with desire but not with behavioral intention. Prior knowledge (through health communications) was significantly related to attitude, desire, and behavioral intention. Overall, the findings support the use of positive anticipated emotions, perceived behavioral condition, prior knowledge, and desire as indicators of participation behavior in the PSC context, and may aid the development of health communication and interventions aimed at encouraging future participation.

## 1. Introduction

Many countries around the world have developed or adopted some types of public sports club systems to promote the health and sociopsychological wellbeing of the general public. The United Kingdom, Germany, Sweden, and Japan are some of the leading countries with highly developed public sport systems that have their governments as the primary source of resources and changes. For example, England came up with the ‘Game Plan’ in 2002 to increase the base of participation in sports, including club sports, using the financial resources from the National Lottery in order to use sports as a vehicle to promote health, quality of life, and social inclusion. South Korea modeled its public sports system after some combinations of German and Japanese public sport systems.

In South Korea, the ‘public sports club’ system was introduced in 2013 with eight clubs by the Department of Culture, Sport and Tourism (DCST), and the number of clubs increased to 72 in 2018, and 92 in 2019 [1,2]. Including those under development (27 clubs), the number of public sports clubs (PSCs) has increased to 125 nationwide as of July 2020 [1,2,3]. Of the 125 clubs, the majority of PSCs are multi-sports clubs (e.g., a PSC offering multiple sport programs), while some of them are sport-specific clubs, such as baseball or ski/snowboard [2]. DCST is planning to make 229 PSCs by the end of 2021 [2,3]. However, DCST’s goal of making 229 PSCs cannot be sustainable without the continued participation of the local residents [4]. In Seoul, Korea, there are currently four multi-sport clubs, while another five PSCs have been approved to be operational in a year or two. Typically, the primary motivations of the PSC members would be accessibility, convenience, and financial aspects (e.g., cheaper or affordable membership fees) due to the government subsidies. 

Currently, a new PSC is initially supported by the central and municipal government for a duration of time (e.g., a three- or five-year support with an option to be extended or discontinued) [2,4]. Consequently, the main issues for any PSCs to be sustainable are recruiting new club members and retaining their current club members. Unfortunately, many PSCs have struggled with encouraging new and sustainable participation [4]. In particular, understanding what influences the current PSC members to continue their participation is much more important to enhance the health-related benefits of the regular physical activity (PA) [5,6,7,8], given the importance of the frequency and duration of PA when it comes to PA as an exercise prescription [7]. On the other hand, 30% of the nation’s population are physically inactive, and only 50% participate in PA more than twice a week, even though the central and local governments have tried to expand the number of public sport facilities, clubs, and sport programs to increase the number of affordable and accessible participation opportunities [1,4].

Despite the superior prediction power of the model of goal-directed behavior (MGB) over its predecessors, such as the theory of planned behavior [9], there have been relatively few attempts to understand individuals’ sport participation behavior using MGB [10,11,12]. Therefore, the current study investigated what influences PSC participants’ continuance intention using an extended MGB. The study integrated individuals’ prior knowledge and awareness through health communications as an additional predictor and investigated the importance of prior knowledge on attitudes, desires, and behavioral intentions. 

### 1.1. MGB Determinants of Behavioral Desire

MGB is one of the consumer decision-making models that extends the theory of reasoned action (TRA) and theory of planned behavior (TPB) [13,14,15], by incorporating desire, anticipated emotions, and the frequency of past behavior [9,10,11,12]. MGB posits that individuals’ behavior can be predicted by their behavioral intentions, perceived behavioral control, and desire, while behavioral desires are driven by their attitudes towards the behavior, subjective norms, positive and negative anticipated emotions, and perceived behavioral condition [9]. In addition, the extended MGB posits that the frequency of past behavior would be positively related to their desires [9]. 

In a similar context, Esposito et al. claimed that MGB could explain a greater variance in physical activity intentions among young adults in comparison to TBP [10]. Their study found that attitudes towards PA, subjective norms and perceived behavioral control were significantly associated with young adults’ desires to participate in PA. Consequently, individuals’ desires were significantly related to intentions towards PA. Similarly, Bavel et al. found that young adults’ desires towards PA were predicted most by attitudes, followed by descriptive norms, perceived behavioral control, and positive anticipated emotions [16]. However, when they were exposed to a positive normative message concerning PA, their desires were mostly predicted by attitudes, followed by positive anticipated emotions, descriptive norms, and negative anticipated emotions. In the context of bicycle touring, Meng and Han found that positive anticipated emotions, subjective norms, perceived behavioral control, the frequency of past behavior, and attitude, in this order, were significantly associated with Chinese bikers’ desires to travel by bike in the near future, while both their desires and the frequency of past behavior were meaningful predictors for their behavioral intentions [17]. For the use of the bicycle for daily travel necessities in a big city, Passafaro et al. found that only positive anticipated emotion and past behavior were statistically significant predictors of bikers’ desires [18]. 

While what statistically predicts behavioral desires varies depending on the contexts, (extended) MGB suggests that volitional desires to perform the activities are guided by attitude, subjective norms, perceived behavioral control, anticipated positive/negative emotions, and past behavior [9,17,19]. For example, an individual with a positive attitude towards participating in a PSC is likely to have a belief that most people around him/her approve of his/her participation, a perception of the ease of participating in a PSC, positive anticipated emotions toward participating in a PSC, and a habit of participating in PSCs (i.e., past behavior) is likely to have a higher level of desires to continuously participate in PSCs. Therefore, the following hypotheses were formulated:

**Hypothesis** **1** **(H1).**  *Attitudes toward PSCs will positively influence (college students’) behavioral desire to participate in PSCs*.

**Hypothesis** **2** **(H2).**  *Subjective norm about participating in PSCs will positively influence behavioral desire to participate in PSCs*.

**Hypothesis** **3** **(H3).**  *Positive anticipated emotions (PAE) will positively influence behavioral desire to participate in PSCs*.

**Hypothesis** **4** **(H4).**  *Negative anticipated emotions (NAE) will positively influence behavioral desire to participate in PSCs*.

**Hypothesis** **5** **(H5).**  *Perceived behavioral control (PBC) will positively influence behavioral desire to participate in PSCs*.

**Hypothesis** **6** **(H6).**  *Frequency of past participation (past behavior) will positively influence behavioral desire to participate in PSCs*.

### 1.2. MGB Determinants of Behavioral Intention

MGB posits that behavioral intentions are the most critical determinants of the corresponding overt behaviors, and they are influenced by desires, PBC, and past behavior [9]. Among these predictors, the desire component is typically the strongest predictor of behavioral intentions compared with PBC and past behavior [9,19]. As mentioned above, Meng and Han suggested that both desires and past behavior were significantly associated with behavioral intentions among bicycle travelers, while the role of PBC on behavioral intentions was not explored in their study [17]. In a meta-analysis in the travel and hospitality context, Chiu and Cho found that desires were the critical antecedent to behavioral intentions, while past behavior was marginally but significantly related to intentions [19]. However, their study did not include PBC as a determinant of behavioral intentions. Kim and Preis claimed that desires and PBC, in this order, were critical components of behavioral intentions, while past behavior did not substantially explain the variance in behavioral intention to use mobile devices [20]. More recently, Qiao et al. found that desires were the most critical predictor of behavioral intentions, followed by PBC and past behavior in the context of tourism [21]. Similarly, Yim and Byon proposed that past satisfaction (through past behavior) was a meaningful predictor of desires and intentions in young adults’ sport consumption decision-making processes [22]. 

Unfortunately, there is no study that investigated desires, PBC, and past behavior all together to understand PA or sport participation. However, the MGB theory and research studies in other contexts strongly suggest that desires, along with PBC and past behavior, are critical determinants of behavioral intentions [9,11,19]. In the context of PSC participation, individuals are likely to have greater behavioral intentions if they have strong desires for PSC participation, positive perceptions about their capacity to participate in PSCs, and habits of participating in PSCs, as a proxy of their positive evaluations on their previous experience. Therefore, the following hypotheses were formed based on the MGB theory:

**Hypothesis** **7** **(H7).**  *Behavioral desire to participate in PSCs will positively influence behavioral intentions to continue participating in PSCs*.

**Hypothesis** **8** **(H8).**  *Perceived behavioral control will positively influence behavioral intentions to continue participating in PSCs*.

**Hypothesis** **9** **(H9).**  *Frequency of past participation will positively influence behavioral intentions to continue participating in PSCs*.

### 1.3. Knowledge and Awareness through Health Communications

The MGB theory can be extended by including additional variables to better understand individuals’ decision-making processes [11,20]. One of the context-specific variables to be included is ‘prior knowledge and awareness of PSCs through health communications [20]. Due to the nature of PSCs, the government agencies and PSCs alike have tried to promote PSCs using various media channels. The fundamental motivation of such health communications, in this case, is to encourage the public’s participation in PSCs and, ultimately, promote health through PA. Kim and Preis suggested that prior knowledge has a positive influence on desires and behavioral intentions to use mobile devices for tourism-related activities, and found that prior knowledge had a stronger influence on behavioral intention than desires [20]. In the context of adolescent participation in new sports, Bae et al. found that prior knowledge was significantly associated with attitude and behavioral intention [23]. Similarly, Park et al. suggested that prior knowledge directly influenced older adults’ attitudes toward exercise and indirectly influenced exercise intentions [24]. As such, it can be posited that individuals with prior knowledge about PSCs and the psychosocial and health benefits associated with participation in PSCs are likely to have a higher level of awareness about PSCs, desires towards PSCs, and intentions to participate in PSCs. Therefore, the following hypotheses were suggested:

**Hypothesis** **10** **(H10).**  *Prior knowledge will positively influence attitudes towards PSCs*.

**Hypothesis** **11** **(H11).**  *Prior knowledge will positively influence behavioral desire to participate in PSCs*.

**Hypothesis** **12** **(H12).**  *Prior knowledge will positively influence behavioral intentions to continue participating in PSCs*.

**Figure 1:**
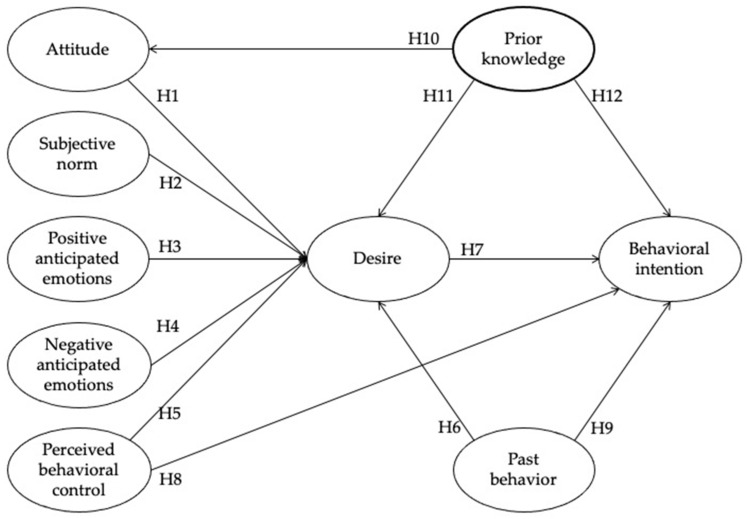
Research model with hypotheses.

Figure 1 summarizes the research hypotheses examined in this study. This research model investigated relationships between attitude, subjective norm, positive and negative anticipated emotion, perceived behavioral control, frequency of past behavior, desire, and behavioral intention, as well as the extended variable, namely prior knowledge (through health communication). 

## 2. Materials and Methods

### 2.1. Participants

Data were collected from 280 college students from four universities (70 students per university) who were enrolled in sports-related elective courses. Specifically, the current study targeted college-aged students who (1) had experience participating in one or more public sport clubs and (2) resided in the Seoul Metropolitan Area using the convenience sampling method. After removing 24 incomplete or invalid responses, a total of 254 completed questionnaires were taken into consideration. Of 254 participants, 68.8% were males (*n* = 176) and 32.2% were females (*n* = 80), and the average age of the respondents was approximately 22 years old (*SD* = 1.78). In terms of monthly participation frequency, the majority of respondents participated in sport clubs fewer than five times per month (*n* = 166, 64.8%), followed by more than 15 times (13.3%), 5~9 times (9.8%), and 10~14 times (12.1%). In terms of participation duration (years), the majority of the respondents have participated in their sport clubs for more than five years (33.2%), followed by 3~4 years (26.6%), 1~2 years (21.1%), and less than one year (19.1%). The most popular sport clubs they have joined include badminton (*n* = 72, 28.1%), swimming (*n* = 58, 22.7%), soccer (*n* = 42, 16.4%), and table tennis (*n* = 31, 12.1%). The rest of the respondents (*n* = 53, 20.7%) are members of various PSCs, such as basketball, tennis, yoga, dance sports, golf, volleyball, baseball, Taekwondo, and rock climbing. Exploratory analysis of variance (ANOVA) found no noticeable differences in the mean scores for study variables based on the type of PSCs. 

So far, there are no detailed statistics available that report the current PSC member profiles. Currently, the only available information is about the number of sport programs and maximum occupancy per sport program at each site. For example, one of the metropolitan PSCs in Incheon reported that this particular site could accommodate up to 380 members across nine sport clubs, including badminton, basketball, tennis, fencing, riflery, table tennis, diving, and curling. A somewhat smaller PSC in Guro, Seoul, could host 210 members across three sports, including badminton, soccer, basketball, and tennis. Therefore, it was deemed that the respondents in this study exhibit a fair representation of the types of sport clubs that the general public typically participate. To ensure no differences across sport programs, ANOVAs on the study variables were conducted. The results show no meaningful differences based on the primary sport program of the respondents.

### 2.2. Instruments

The MGB constructs were taken from previous studies applying MGB to consumer studies [9,10,11,12,13,14,16,20]. The survey instrument included the following sub-groups of variables, mainly from the MGB constructs: attitudes towards PSCs, subjective norm, perceived behavioral control (PBC), positive anticipated emotions (PAE), negative anticipated emotions (NAE), desire, prior knowledge through health communication, and intention to continue participating in PSCs, as well as demographic variables such as age, gender, and PSC-related questions (e.g., participation frequency). 

All items, excluding demographic variables such as gender and the frequency of past behavior, were measured with three or four items and by means of a five-point Likert-type scale, ranging from 1 (strongly disagree) to 5 (strongly agree). Examples of the scale items are: “participating in PSCs is something that makes me feel good” (attitude), “most of the other people important to me think I should participate in PSCs” (subjective norm), “it is mostly up to me whether I participate in PSCs in the next week” (PBC), “If I participate in PSCs, I will feel excited” (PAE), “If I do not participate in PSCs, I will be disappointed” (NAE), “I have enough knowledge about PSCs that I have learned from health communication” (prior knowledge), “how often did you participate in PSCs during the last month?“ (frequency of past behavior), “I am eager to participate in PSCs” (desire), and “I am planning to participate in PSCs in the near future” (intention). 

### 2.3. Statistical Analysis

Initially, descriptive and correlation analyses were conducted to examine the normality assumption of the data, including skewness and kurtosis statistics, and found no major issues. Data were primarily analyzed using the two-step procedure for structural equation modeling (SEM) [25]. Firstly, confirmatory factor analysis (CFA) was performed to examine the measurement model in terms of reliability and validity of the measures. Secondly, SEM was conducted to test the relationships between study variables. The software used was SPSS 25 and AMOS 25. 

## 3. Results

### 3.1. Measurement Model Assessment

CFA assessed the psychometric properties of the measurement model. The results show that the measurement model fitted the data well: χ^2^ (377) = 740.12, χ^2^/*df* = 1.96, CFI = 0.95, TLI = 0.94, RMSEA = 0.06 [26,27,28,29,30]. The reliability of the measures was evaluated by examining Cronbach’s alpha coefficients and composite reliability (CR) values (see Table 1) [30,31]. The results show that the scales exhibited good reliability as the Cronbach’s alpha coefficients of all constructs were more than acceptable, ranging from 0.72 to 0.95 [31]; and all the CR values varied from 0.77 to 0.97, thus fulfilling the threshold criterion (>0.70) [30]. The construct and convergent validity of the measures was examined using factor loadings, and average variance extracted (AVE) values [26,27,28,29,30]. Due to the double-loading, one item from the ‘intention’ scale was removed. All factor loadings of the measures were highly significant, and the smallest factor loading was 0.68. The AVE values were greater than 0.50 (ranged from 0.52 to 0.88), fulfilling the threshold criteria. Overall, the measurement model demonstrated adequate psychometric properties. 

### 3.2. Structural Model Assessment

The results indicate that the proposed structural model fitted the data reasonably well, χ^2^ (444) = 1,113.66, χ^2^/*df* = 2.51, CFI = 0.92, TLI = 0.91, RMSEA = 0.077 [28]. Table 2 reports the results of the standardized path coefficients for all hypothesized relationships between variables. 

Of the six MGB constructs in predicting desire to participate in PSCs, positive anticipated emotion was the most significant determinant (*β* = 0.46, *p* < 0.001), followed by PBC (*β* = 0.40, *p* < 0.001) and past behavior (*β* = 0.11, *p* < 0.01), thus supporting H3, H5 and H6. However, other MGB predictors, namely attitude, subjective norm, and negative anticipated emotion, were not significant, thus rejecting H1, H2, and H4. Moreover, intention (to continue participating in PSCs) was significantly associated with desire (*β* = 0.68, *p* < 0.001) but not with PBC and past behavior, thus supporting H7 but rejecting H8 and H9. 

**Figure 2:**
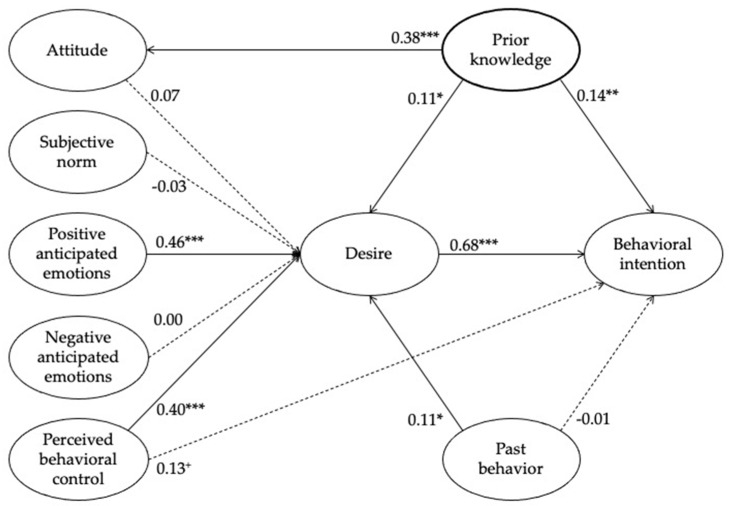
Research model with path coefficients. Note: * *p* < 0.05, ** *p* < 0.01, *** *p* < 0.001, + *p* < 0.10.

In addition, the effects of the extended variable on attitude, desire, and intention were examined. Prior knowledge (through health communication) was significantly associated with attitude (*β* = 0.38, *p* < 0.001), desire (*β* = 0.11, *p* < 0.05), and future intention (*β* = 0.14, *p* < 0.01), thus supporting H10, H11, and H12. Overall, the results indicate that prior knowledge of PSCs (and subsequent psychosocial and health benefits) contributes to the increased likelihood of continued participation in PSCs. Figure 2 illustrates the relationships between variables based on the SEM results. 

## 4. Discussion

The purpose of the current empirical study was to investigate what influences the decision-making process of the PSCs users using the extended MGB framework and also to explore the role of prior knowledge and awareness gained through heath communications. The study found that positive anticipated emotions, perceived behavioral control, prior knowledge, and past behavior, in that order, were associated with PSC members’ desires, whereas their desires and prior knowledge were significantly related to behavioral intentions to continue. Therefore, as desires, positive anticipated emotions, PBC, prior knowledge, and the frequency of past behavior increase, the likelihood of participation in PSCs increases [9,10,16]. This suggests that in the PSC context where continued and frequent participation is a primary goal to enhance physical health and socio-psychological wellbeing, health and human services agencies (including PSC managers) should prioritize the ways to enhance potential and current PSC participants’ anticipation emotions, increase their behavioral control (or remove participation barriers), and promote knowledge and awareness about PSCs and associated benefits. In doing so, health agencies and PSC managers can enhance participants’ behavioral desires to (re)participate in PSCs and, thus, PA. More detailed strategies to foster participants’ behavioral desires were discussed below. 

This study found that positive anticipated emotion greatly influences college-aged individuals’ desire to (continue to) participate in PSCs, consistent with the findings of Bavel et al., Meng and Han, and Chiu and Cho [16,17,19]. Current and potential PSC members with positive feelings about participating in PSCs for health and social purposes are likely to have a strong desire to participate in PA through PSCs. As discussed previously, a meta-analysis in the context of travel and tourism found that the emotional factors, especially perceived anticipated emotions, were the most significant antecedents of desire in comparison to other cognitive (i.e., attitude and subjective norms), perceptive (i.e., PBC), and habitual factors (i.e., frequency of past behavior) [19]. 

Participating in PA or PSCs can be considered as a way to improve participating individuals’ health and, thus, considered a utilitarian behavior. However, it seems that individuals are more likely to participate in PSCs for the hedonic aspects of PSC-related experience due to the nature of PSCs (e.g., practicing and playing with others). PSC members’ primary motivation could be more about, for example, having a good time with others and releasing ones’ stress through sport activities. The result of the relationship between the expectation of negative emotions and desire corroborates the importance of perceived anticipated emotions to PSC participants in terms of their desires and behavioral intentions [19]. Additionally, the concept of enjoyment should be well embraced. For example, Gardner et al. found that participants’ level of enjoyment in their main sport was one of the most critical predictors of sport participation and dropout in the context of organized sports [5]. 

Consequently, PSC managers develop strategies to create a fun and enjoyable environment for their participants, while health agencies must develop communication strategies to emphasize the hedonic benefits of the PSC participation as opposed to the utilitarian benefits. In particular, PSC managers should refer to the service quality literature. For example, Ko and Pastore suggested that sport center managers should improve their PSC’s service quality by enhancing their service offerings in the following four dimensions: program quality (e.g., range of program, operating time, and information), interaction quality (e.g., client-employee interaction and inter-client interaction), outcome quality (e.g., physical change, valence, and sociability), and environment quality (e.g., ambient condition, design, and equipment) [32]. 

This study also found that desire was significantly influenced by the nonvolitional factor (i.e., PBC) and the habitual factor (i.e., the frequency of past behavior). PSC members are likely to have a strong desire to engage in a specific behavior (e.g., revisitation) when they positively evaluate their situation and self-efficacy in a given situation (i.e., PBC) [9]. Thus, health agencies and PSC managers should develop strategies to reduce or minimize any perceived barriers to PSC participation by referring to the concepts of leisure constraints and facilitators. Leisure constraints are factors that inhibit our ability to participate in leisure activities while facilitators to leisure are factors that encourage or enhance leisure participation [33]. Initially, they need to identify what negatively influences individual participation in PSCs (i.e., identifying participation constraints). Once identifying major participation constraints and barriers, they need to think about mitigation strategies (i.e., affordance and facilitators) to enhance the level of individual PBC [33]. 

In addition, (the frequency of) past behavior was a significant predictor of desire. It has been well documented that consumers’ experience would influence their decision-making process by integrating previous beliefs and attitudes with new information [19,34] and becoming familiar with a specific behavior (e.g., participating in PSCs) [19]. Thus, PSC managers should make their users’ first visit a pleasant experience. 

Lastly, the current study found the meaningful role of prior knowledge via health communications [20]. As hypothesized, prior knowledge about PSCs and information about the benefits associated with PSC participation was significantly associated with (positive) attitudes toward PSCs, their desires to re-participate, and behavioral intentions. Fairly often, the messages conveyed by health communications tend to focus on health benefits (i.e., utilitarian motivations). In our study’s context, it makes more sense to develop more hedonic-oriented messages to encourage potential and current PSC members’ (re)participation to change their attitudes towards PSCs and desire and intentions to participate in PSCs. 

Unlike our hypotheses, the current study did not find meaningful relationships between volitional/cognitive factors (i.e., attitude and subjective norms) and desire. It might be due to the fact that, in general, the public is well aware of the PSCs and their functions. From a different angle, this no-influence of attitudes on desires indirectly supports the importance of emotional antecedents on desires. However, this result should be interpreted with caution. In other national or regional contexts (e.g., a country with a low PA level), it is possible that, as the MGB theory posits, attitudes could be one of the most critical antecedents to PA or PSC participation [19]. 

There are several suggestions for future studies. Firstly, as mentioned above, future studies should consider cultural and contextual differences. For example, certain countries may have different perceptions and beliefs about the role of PA on their health and psychosocial wellbeing. It is also possible that certain countries or regions within a nation may come with varying levels of constraints and barriers to PA [11,33]. Secondly, the current study utilized a cross-sectional research design. Thus, future studies could benefit from different research designs such as a longitudinal study. Thirdly, researchers may consider exploring individual differences and preferences such as preferred types of sport activities and gender differences among different age groups [8]. Lastly, the current study primarily used the MGB as a conceptual framework to understand what influences college-aged students’ participation in PSCs. In doing so, the present study was not able to incorporate other perspectives or variables (e.g., a functional approach; motivational profiles). Therefore, future studies should consider meaningful extended variables to be included in the MGB framework. 

## 5. Conclusions

In Korea, the central and local governments have aimed to increase the number of PSCs as a way to promote national health and enhance the public’s sociopsychological wellbeing. Unfortunately, the number of PSC participants, especially in terms of repeat participation, has been less than satisfactory. The results of the current study suggest that health and human service agencies in Korea should focus on emotional elements (i.e., positive anticipated emotions) and nonvolitional elements (i.e., PBC) to develop desires to participate in PSCs. In doing so, health communications need to convey not only health-related information but also hedonic-oriented information to encourage the public’s PSC participation. 

## Figures and Tables

**Table 1:** Correlations, reliability and validity.

Variable	1	2	3	4	5	6	7	8	*α*	AVE	CR
1. Attitude	1								0.90	0.74	0.92
2. Subjective norm	−0.06	1							0.72	0.52	0.77
3. PBC	0.51 **	−0.14 *	1						0.95	0.78	0.93
4. PAE	0.70 **	−0.08	0.66 **	1					0.95	0.85	0.96
5. NAE	0.12	−0.23 **	0.17 **	0.18 *	1				0.95	0.88	0.97
6. Desire	0.62 **	−0.13	0.75 **	0.77 **	0.15 *	1			0.95	0.87	0.95
7. Intention	0.61 **	−0.01	0.66 **	0.87 **	0.14 *	0.78 **	1		0.93	0.82	0.93
8. Prior knowledge	0.28 **	−0.02	0.26 **	0.32 **	−0.04	0.37 **	0.40 **	1	0.85	0.58	0.84
9. Past behavior	0.27 **	−0.10	0.33 **	0.26 **	−0.02	0.37 **	0.35 **	0.24 **	−	−	−
Mean	3.57	3.43	2.99	3.41	1.91	3.21	3.18	2.97			
S.D.	0.82	0.73	1.07	0.87	0.71	1.00	0.94	0.84			

Note 1: * *p* < 0.05, ** *p* < 0.01; PBC = perceived behavioral control, PAE = positive anticipated emotions, NAE = negative anticipated emotions, α = Cronbach’s alpha, AVE = average variance extracted, CR = composite reliability. Note 2: Other potential correlates (age, gender, and the duration of participation) were excluded due to non-significant results.

**Table 2:** Results of the hypothesized model.

Hypothesized Paths	*β*	Standard Error	*t*-Value
H1. Attitude → Desire	0.07	0.05	1.72
H2. Subjective norm → Desire	−0.03	0.03	−0.74
H3. PAE → Desire	0.46	0.06	8.07 ***
H4. NAE → Desire	0.001	0.05	0.02
H5. PBC → Desire	0.40	0.05	7.37 ***
H6. Past behavior → Desire	0.11	0.03	3.00 **
H7. Desire → Intention	0.68	0.07	8.57 ***
H8. PBC → Intention	0.13	0.06	1.85
H9. Past behavior → Intention	−0.01	0.03	−0.21
H10. Knowledge → Attitude	0.38	0.08	5.17 ***
H11. Knowledge → Desire	0.11	0.06	2.25 *
H12. Knowledge → Intention	0.14	0.06	2.85 **

Note: * *p* < 0.05, ** *p* < 0.01, *** *p* < 0.001

## Data Availability

The data that support the findings of this study are available on reasonable request from the corresponding author at khh@honam.ac.kr.
